# Evaluation of sorafenib for advanced hepatocellular carcinoma with low α-fetoprotein in arrival time parametric imaging using contrast-enhanced ultrasonography

**DOI:** 10.1007/s10396-016-0757-2

**Published:** 2016-11-11

**Authors:** Kazue Shiozawa, Manabu Watanabe, Takashi Ikehara, Ryo Shimizu, Mie Shinohara, Yoshinori Igarashi, Yasukiyo Sumino

**Affiliations:** Division of Gastroenterology and Hepatology, Department of Internal Medicine, Toho University Medical Center, Omori Hospital, 6-11-1 Omorinishi, Ota-ku, Tokyo, 143-8541 Japan

**Keywords:** Hepatocellular carcinoma, Sorafenib, Contrast-enhanced ultrasonography, Arrival time parametric imaging, α-Fetoprotein

## Abstract

**Purpose:**

To determine the usefulness of arrival time parametric imaging (AtPI) using contrast-enhanced ultrasonography (CEUS) with Sonazoid in evaluating early response to sorafenib for hepatocellular carcinoma (HCC).

**Methods:**

Twenty-one advanced HCC patients with low α-fetoprotein (AFP) levels (≤35 ng/ml) who received sorafenib for at least 4 weeks were enrolled in this study. CEUS was performed before and 2 weeks after treatment, and the images of the target lesion in the arterial phase were analyzed by AtPI. In the color mapping images obtained by AtPI, the mean arrival time of the contrast agent in the target lesion from the reference point (mean time: MT) was calculated. In each patient, differences between MT before and MT 2 weeks after treatment were compared. MT (+) and MT (−) groups were defined as difference of 0 s or greater and less than 0 s, respectively. Overall survival was evaluated between the two groups.

**Results:**

In the MT (+) (11 patients) and MT (−) (10 patients) groups, the median survival time was 792 and 403 days, respectively, which was statistically significant.

**Conclusions:**

The results suggested that AtPI was useful for evaluating early response to sorafenib for advanced HCC with low AFP level.

## Introduction

Sorafenib (Nexavar; Bayer Healthcare, Leverkusen, Germany) is an oral multi-targeted tyrosine kinase inhibitor that is indicated for unresectable advanced hepatocellular carcinoma (HCC) and significantly improves progression-free and overall survival [1]. Thus, sorafenib is widely used for treatment of unresectable advanced HCC, but it is also an expensive drug that has certain adverse events [1]. Therefore, evaluation of the early response to sorafenib is required for patients to continue treatment with the drug. Sorafenib has antitumor effects that include tumor growth inhibition and antiangiogenic effects, which make it challenging to evaluate its therapeutic effects using the conventional Response Evaluation Criteria in Solid Tumors (RECIST) [2]. Alternative evaluation criteria, including tumor necrosis and intratumor hemodynamics, such as the modified RECIST (mRECIST) [3], Response Evaluation Criteria in Cancer of the Liver (RESICL) [4], and Choi criteria [5], have been recommended.

We have investigated the therapeutic effects of sorafenib for advanced HCC using arrival time parametric imaging (AtPI) [6] with contrast-enhanced ultrasonography (CEUS) using Sonazoid (Daiichi Sankyo, Tokyo, Japan), which is a tool to evaluate the delay in the arrival of the contrast agent at the region of interest (ROI) compared with that at the reference point. Our results suggest that AtPI may be useful for early evaluation of therapeutic responses to sorafenib in patients with advanced HCC [7]. Serum α-fetoprotein (AFP) [8–11], des-γ-carboxy prothrombin (DCP) [12], vascular endothelial growth factor (VEGF) [13], and neutrophil–lymphocyte ratio (NLR) [14] have been investigated as therapeutic biomarkers of sorafenib. AFP is a particularly useful prognostic factor for sorafenib because of its simplicity of measurement. In clinical settings, however, the AFP level is low in some cases of advanced HCC [1]. In this study, we examined the utility of AtPI with CEUS for early evaluation of the therapeutic effect of sorafenib for advanced HCC with a low AFP level.

## Materials and methods

Of 125 patients with advanced HCC in whom sorafenib treatment was initiated at our hospital between April 2009 and December 2015, 21 who met the following criteria were selected retrospectively: (1) consent to this study, (2) AFP ≤35 ng/ml before administration, and (3) CEUS performed before and 2 weeks after administration. The patients included 18 males and three females, and the mean age was 71.0 years old (50–84 years old). The underlying liver disease was hepatitis B in two patients, hepatitis C in 12, alcoholic hepatitis in four, and non-alcoholic steatohepatitis in three. The Child–Pugh classification was A in 16 and B in five. Before administration, the median AFP level was 11.7 ng/ml (1.7–34.8 ng/ml), the median DCP level was 149 mAU/ml (17–60,347 mAU/ml), and the median neutrophil–lymphocyte ratio (NLR) was 2.1 (1.2–6.3). Transarterial infusion (TAI), transarterial chemoembolization (TACE), persistent hepatic transarterial infusion chemotherapy (HAIC), and no treatment were used after sorafenib treatment in one, three, eight, and nine patients, respectively. For TAI, cisplatin (IA-call^®^; Nippon Kayaku, Tokyo, Japan) was administered via a catheter; for TACE, Farmorubicin^®^ (Pfizer, Tokyo, Japan), Lipiodol^®^ (Laboratoire Guerbet, Aulnay-Sous-Bois, France), and 1-mm Gelpart^®^ (Nippon Kayaku, Tokyo, Japan) were administered via a catheter; and for HAIC, daily cisplatin (10 mg/body on days 1–5) given over 1 h and 5-fluorouracil (5-FU) (250 mg/body on days 1–5) given over 23 h were infused every 4 weeks via an implantable port system (Table 1). The initial dose of sorafenib in all patients was 400 mg/day. Following the Evidence-based Clinical Practice Guidelines for HCC developed by the Japan Society of Hepatology (JSH) [15], patients were diagnosed with advanced HCC based on the presence of ≥4 HCC lesions or portal vein tumor thrombus (PVTT) with ≥Vp3 on dynamic computed tomography (CT) or abdominal angiography.

**Table 1:** Characteristics of all patients (*n* = 21)

Variables	All lesions (*n* = 21) (range)
Treatment duration, days (median)	111 (range 15–443)
Age, years (median)	71 (range 50–84)
Gender	
Male/female	18/3
Etiology	
HBV/HCV/alcohol/NASH	2/12/4/3
Child–Pugh classification	
A/B	16/5
Previous treatment y/n	19 (TACE/RFA:17/14)/2
Post-treatment, TAI or TACE/HAIC/none	4/8/9
BCLC, B/C	12/9
Vascular invasion, y/n	4/17
Extrahepatic metastasis, y/n	5/16
AFP, ng/mL (median)	11.7 (range 1.7–34.8)
DCP, mAU/mL (median)	149 (range 17–60,347)
NLR	2.1 (1.2–6.3)

*HBV* hepatitis B virus, *HCV* hepatitis C virus, *NASH* non-alcoholic steatohepatitis, *RFA* radiofrequency ablation, *TACE* transarterial chemoembolization, *TAI* transarterial infusion, *HAIC* persistent hepatic transarterial infusion chemotherapy, *BCLC* Barcelona Clinic Liver Cancer, *AFP* alpha-fetoprotein, *DCP* des-γ-carboxy prothrombin, *NLR* neutrophil to lymphocyte ratio

CEUS was performed before and 2 weeks after sorafenib administration [7]. One lesion that could be followed for a period within 10 cm from the liver surface was selected using ultrasonography in each patient to standardize evaluations, and CEUS was performed in the same cross-section and under the same conditions at all time points. The ultrasound equipment used in this examination was SSA-790A (Toshiba Medical Systems, Tokyo, Japan) with a convex probe (PVT-375BT, 3.75-MHz center frequency). The imaging mode used was wideband harmonic imaging (pulse subtraction) with transmission/reception frequencies of 1.8 and 3.5 MHz, respectively. The mechanical index for acoustic output was set to 0.2, and the dynamic range was set to 60–65 dB. A single focus point was set at the deep site of the lesion, and a bolus intravenous injection of Sonazoid (0.5 ml) was administered via a left cubital venous line, followed by a 10-ml normal saline flush. After injection of Sonazoid, the patients were asked to hold their breath. The vascular phase (0–40 s) was observed, and video images were recorded and analyzed by an offline procedure using AtPI.

**Fig. 1:**
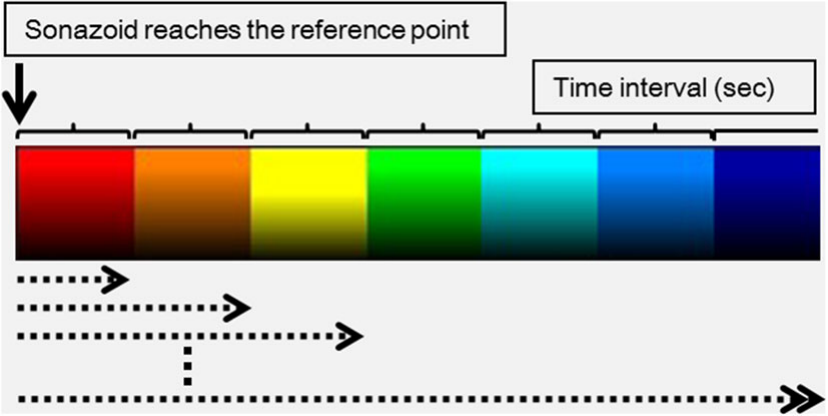
Delays in the arrival of the contrast agent at the target site compared with that at the reference point (0 s) are represented by *red*, *orange*, *yellow*, *green*, *light blue*, *blue*, and *navy blue* at 0.5 s intervals

AtPI was performed using image analysis software for Aplio/Xario (Toshiba Medical Systems, Tokyo, Japan), based on the report by Watanabe et al. [6]. In this method, an appropriate site, such as an intrahepatic artery or tumor vessel, is identified as a reference point, and the time at which the contrast agent reaches this site is defined as the zero point. Differences in arrival times at the target lesion from reference points are determined on the diagnostic images, and these time differences are color mapped. In this study, the moment of arrival of the contrast agent at a large artery near the tumor was regarded as the reference point. In color mapping, delays in the arrival of the contrast agent at the target site compared with that at the reference point (0 s) are represented as red → orange → yellow → green → light blue → blue → navy blue at 0.5 s intervals (Fig. 1). In prepared color mapping images, a maximum ROI was determined for each lesion, and the mean arrival time of the contrast agent in the ROI from the reference point (mean time; MT) was calculated. MT was measured three times in each patient, and the mean value was used. In each patient, differences in the MT 2 weeks after initiation of treatment were compared with the MT before treatment. Blood flow velocity was judged to have been reduced when the time difference was zero or greater (MT (+) group), and to have been increased when the difference was less than zero (MT (−) group). All MT yielded by AtPI were evaluated by a hepatologist with 15 years of experience.

Analysis 1: Cumulative survival rates were compared between the MT (+) and MT (−) groups using the Kaplan–Meier method, and the significance of differences was analyzed by log-rank test. Survival rates were calculated from the start of sorafenib administration to final follow-up or death.

Analysis 2: Ten background factors—mean age, gender (male/female), administration period, Child–Pugh classification (A/B), presence or absence of previous treatment, Barcelona Clinic Liver Cancer (BCLC) classification (B/C) [16], presence or absence of PVTT (Vp 3–4) and/or hepatic vein invasion (Vv), presence or absence of extrahepatic metastasis, DCP level, and NLR—were compared between the two groups by χ^2^ test, Fisher exact test, Student *t* test, and Mann–Whitney *U* test.

Analysis 3: Eight factors—MT (+/−), age (≥70 or <70 years), Child–Pugh classification (A/B), BCLC classification (B/C), presence or absence of PVTT (Vp 3–4) and/or Vv, presence or absence of extrahepatic metastasis, DCP level (≥100 or <100 mAU/ml), and NLR (≥2.5 or <2.5)—were examined in univariate analysis using a Cox hazard model and multivariate analysis using the stepwise forward selection method.

Statistical analysis was performed using SPSS version 11.0 for Windows (SPSS, Inc., Chicago, IL, USA). *p* < 0.05 was considered to indicate a significant difference.

## Results

**Fig. 2:**
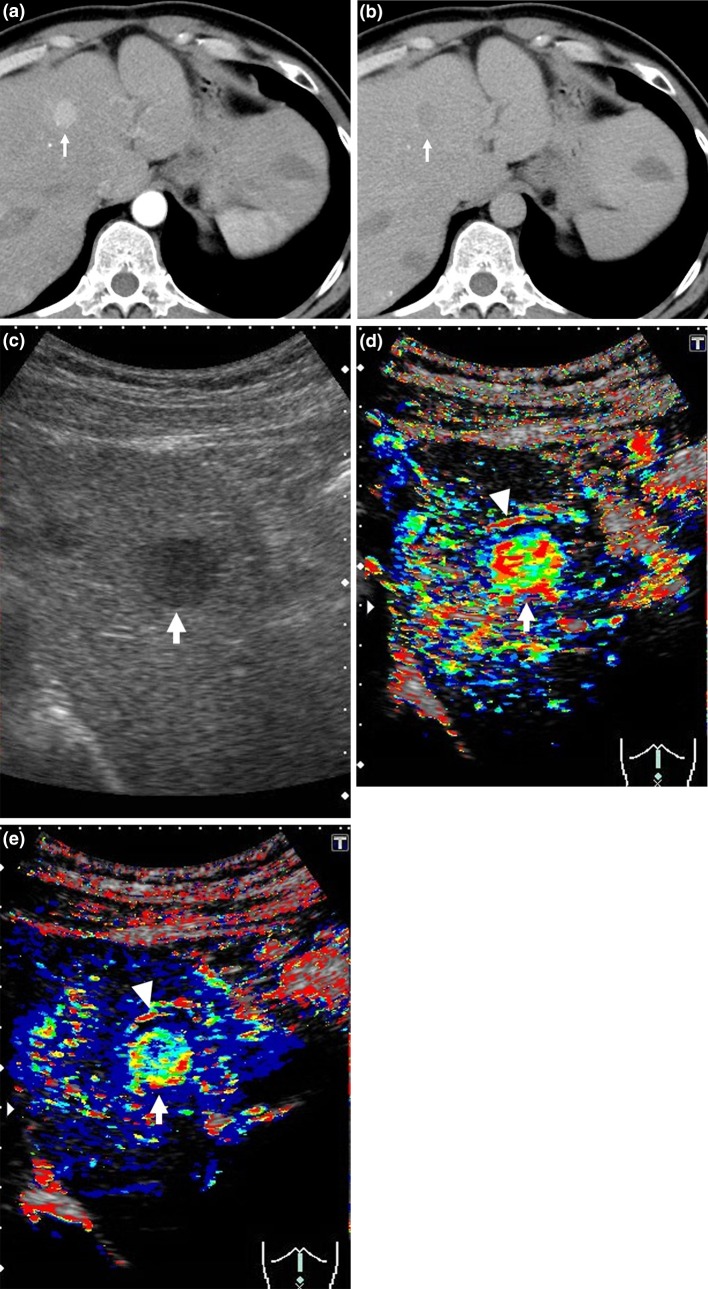
Clinical example of MT (+) group. The patient was a 50-year-old male with chronic hepatitis B virus. Sorafenib administration (400 mg/day) was started for advanced hepatocellular carcinoma (HCC). **a** Dynamic computed tomography (CT) scan in arterial phase before treatment showed a hypervascular lesion measuring 18 mm in diameter in S4 (*arrow*), which was the target lesion. **b** Dynamic CT scan in equilibrium phase before treatment showed a hypoattenuating lesion in S4 (*arrow*). **c** Gray-scale ultrasonography showed a low echoic tumor measuring 18 mm in diameter in S4 (*arrow*). This tumor was established as a target lesion. **d** The *color* mapping image before treatment showed primarily *red*, *yellow*, or *green* in the tumor (*arrow*). A large artery near this tumor was regarded as the reference point (*arrow head*). **e** The *color* mapping image 2 weeks after treatment showed primarily *light blue* or *blue* in the tumor (*arrow*). The same artery before treatment was regarded as the reference point (*arrow head*)

**Fig. 3:**
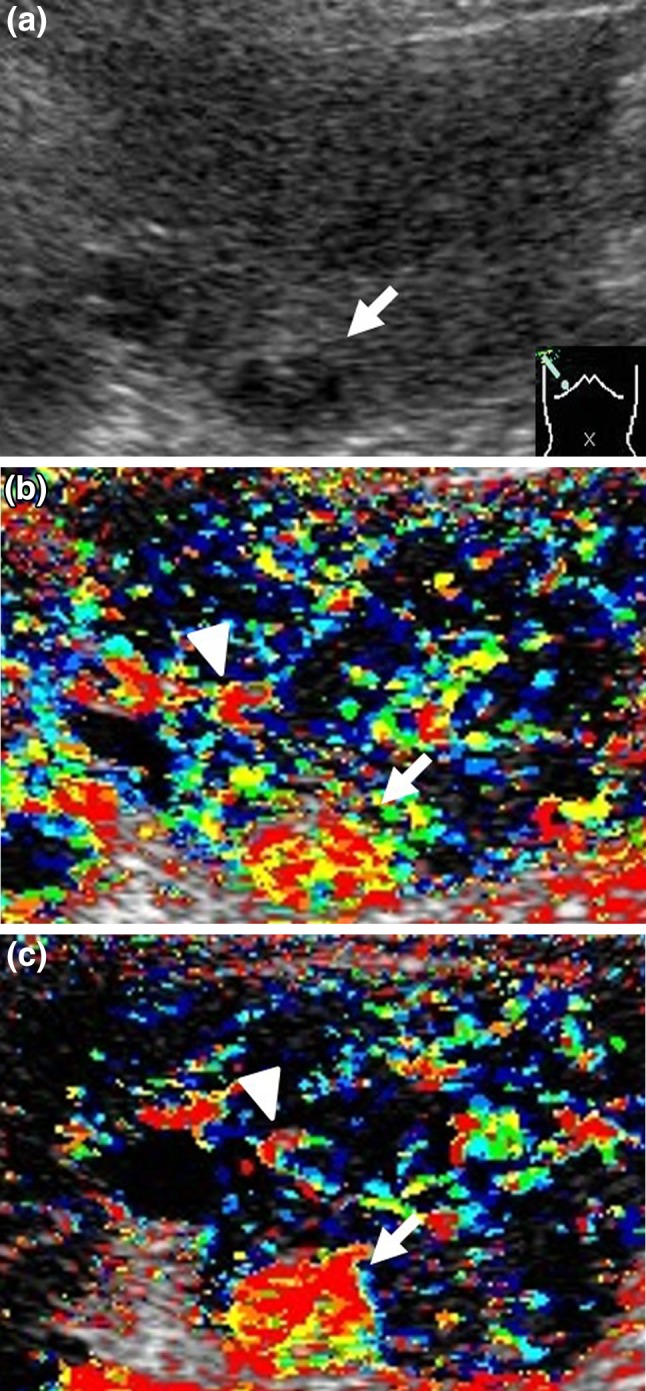
Clinical example of MT (−) group. The patient was a 71-year-old male with chronic hepatitis C virus. Sorafenib administration (400 mg/day) was started for advanced hepatocellular carcinoma (HCC). **a** Gray-scale ultrasonography showed a low echoic tumor measuring 16 mm in diameter in S6 (*arrow*). This tumor was established as a target lesion. **b** The color mapping image before treatment showed primarily* red* or* yellow* in the tumor (*arrow*). A large artery near this tumor was regarded as the reference point (*arrow head*). **c** The color mapping image 2 weeks after treatment showed primarily red in the tumor (*arrow*). The same artery before treatment was regarded as the reference point (*arrow head*)

**Fig. 4:**
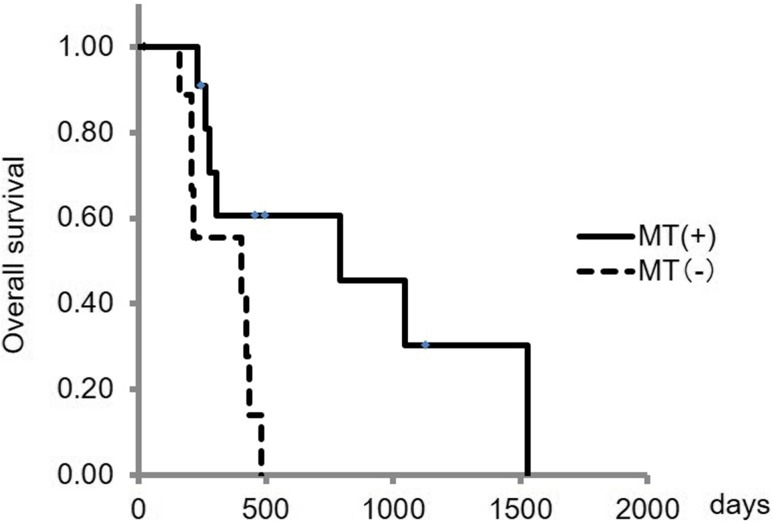
Comparison of cumulative overall survival in the MT (+) and MT (−) groups

The MT (+) (Fig. 2) and MT (−) (Fig. 3) groups included 11 and 10 patients, respectively. The median survival times in these groups were 792 and 403 days, respectively, with significant prolongation of OS in the MT (+) group (*p* = 0.014) (Fig. 4). In a comparison of background factors between the two groups (Table 2), NLR was significantly higher in the MT (−) group (*p* = 0.01). The prognostic factors were a MT (−) status (hazard ratio (HR) 4.13, 95% CI 1.22–13.98, *p* = 0.02) and high NLR (HR 3.30, 95% CI 1.04–10.45, *p* = 0.04) in univariate analysis, and a MT (−) status (HR 3.67, 95% CI 1.08–12.46, *p* = 0.04) in multivariate analysis (Table 3).

**Table 2:** Patient characteristics in the MT (+) and MT (−) groups

	MT (+) (*n* = 11)	MT (−) (*n* = 10)	*p*
Age, years (median)	68	73.5	0.46
Sex, M/F	11/0	7/3	0.09
Treatment duration, days (median)	111	117	1.00
Child–Pugh classification, A/B	9/2	7/3	0.64
Previous treatment, y/n	10/1	9/1	1.00
BCLC, B/C	7/4	5/5	0.67
Vascular invasion, y/n	3/8	1/9	0.59
Extrahepatic metastasis, y/n	1/10	4/6	0.15
DCP, mAU/mL (median)	102	94	1.00
NLR (median)	1.97	2.7	0.01

*BCLC* Barcelona Clinic Liver Cancer, *DCP* des-γ-carboxy prothrombin, *NLR* neutrophil to lymphocyte ratio

**Table 3:** Prognostic factors in all patients

Variable	Univariate analysis				Multivariate analysis			
	HR	95% CI		*p*	HR	95% CI		*p*
MT, +/−	4.13	1.22	13.98	0.02	3.67	1.08	12.46	0.04
Age, years (<70/≥70)	1.39	0.48	4.04	0.54				
Child–Pugh classification, A/B	0.59	0.13	2.75	0.51				
BCLC, B/C	1.83	0.57	5.84	0.31				
Vascular invasion, y/n	1.04	0.23	4.79	0.96				
Extrahepatic metastasis, y/n	2.10	0.60	7.31	0.24				
DCP, mAU/mL (<100/≥100)	0.86	0.30	2.48	0.77				
NLR (<2.5/≥2.5)	3.30	1.04	10.45	0.04				

*BCLC* Barcelona Clinic Liver Cancer, *DCP* des-γ-carboxy prothrombin, *NLR* neutrophil to lymphocyte ratio

## Discussion

The antitumor effect of sorafenib for advanced HCC can be evaluated based on disappearance or reduction of intense tumor staining, which reflects ischemic changes, on contrast-enhanced CT and magnetic resonance imaging [17]. The high temporal resolution of CEUS allows microhemodynamics to be evaluated, and this may be useful for assessment of the effect of sorafenib treatment. In our previous study of 14 patients with advanced HCC treated with sorafenib, MT values of treatment response obtained using AtPI at 2 and 4 weeks after treatment were compared with mRECIST-based criteria on dynamic CT performed at 4–8 weeks after treatment. MT differed significantly between cases with stable disease-partial response (SD-PR) and progressive disease (PD) at 2 and 4 weeks after treatment and was mostly consistent with the mRECIST-based evaluation of the treatment response. These results suggest that MT using AtPI is useful for early evaluation of the treatment effect of sorafenib [7].

Serum biomarkers that are simple to measure, such as AFP [8–11] and DCP [12], may also be useful to evaluate the treatment response and predict the outcome of sorafenib for advanced HCC. Several studies have examined AFP as a predictive biomarker for sorafenib. OS is significantly prolonged in patients in whom the AFP level decreases by 20% at 2–4 or 8 weeks after treatment compared with that before treatment [8, 9]. In another study, the AFP level increased significantly at 2 weeks after treatment compared to before treatment in cases with PD [10]. Nakazawa et al. [11] reported that an increase in AFP of ≥20% within 4 weeks after treatment compared with before treatment indicated a poor prognosis.

These results suggest that AFP may serve as a prognostic factor in sorafenib treatment; however, the AFP level is low in some cases of advanced HCC. For example, in the SHARP study [1], AFP was <20 ng/ml in 34.6% of 602 patients. In this study, therefore, we used AtPI with CEUS for patients with advanced HCC with a low AFP level to compare MT before and after treatment and evaluate the utility of this parameter for early evaluation of the treatment effect. OS was significantly prolonged in the MT (+) group (i.e., blood flow velocity of the lesion was reduced after treatment), compared to the MT (−) group (i.e., blood flow velocity of the lesion was increased after treatment), and NLR was significantly higher in the MT (−) group.

Aggravation of nutritional conditions and chronic inflammatory reactions are involved in disease progression in patients with cancer. Thus, inflammation-based prognostic scores and NLR, a systemic marker of inflammation, may serve as prognostic factors in various cancers, including stomach, colorectal, and non-small cell lung cancer [18]. In a study in 132 patients with HCC treated by transcatheter arterial embolization, the prognosis was poor in those with a high NLR [19]. Mechanistically, neutrophils release chemokines and promote vascularization, which promotes tumor growth, whereas lymphocytes inhibit tumor growth because they are involved in antitumor immunity [20, 21]; therefore, an increase in neutrophils and a decrease in lymphocytes (i.e., an increase in NLR) are associated with tumor progression, which may aggravate the prognosis. In this study, NLR was significantly higher in the MT (−) group, suggesting a correlation between MT and NLR. This finding also suggests that MT is a useful index for evaluation of the treatment effect of sorafenib. MT (−) status and high NLR were prognostic factors in univariate analysis, and MT (−) status was the only independent factor in multivariate analysis, suggesting that MT may be more useful than NLR for evaluation of the treatment effect of sorafenib.

Kuzuya et al. [17] suggested that ischemic changes observed in imaging 2 weeks after sorafenib treatment reflected the treatment effect and could serve as a prognostic factor. Similarly, in the current study, evaluation was performed 2 weeks after treatment, and it is desirable to evaluate the response to sorafenib as early as possible because of the characteristics of the drug. Our results suggest that early evaluation of the treatment effect of sorafenib using AtPI is useful in patients with advanced HCC with a low AFP level.

As limitations, we note that the study was performed in a small number of patients and only one lesion was investigated. However, previous studies of treatment effects using CEUS have yielded significant findings based on a single target lesion [7, 22], which suggests that evaluation of the treatment effect of sorafenib based on a single target using AtPI is acceptable. It is possible MT may be affected by changes in the hemodynamics of peritumoral hepatic parenchyma. However, the impact of the background hepatic hemodynamics is likely to be small because the time required for the contrast agent to reach the tumor from the reference point, which was an artery near the tumor, was compared in individual subjects in this study.

And not all patients could be examined by dynamic CT before and after treatment because patients with renal impairment and iodine allergy were included in this study. Therefore, only a small number of patients could be investigated, and the time to progression was not evaluated. Since CEUS is noninvasive, we are planning to increase the number of cases and continue to investigate AtPI using CEUS as a biomarker for sorafenib treatment.

## Conclusion

We suggest that changes in MT on AtPI using CEUS are useful to evaluate the treatment effect of sorafenib in patients with advanced HCC with a low AFP level. Determination of MT as a biomarker may allow early evaluation of the response to sorafenib treatment.
